# Editorial: Recent advances in small molecule-based targeted therapy for breast cancer

**DOI:** 10.3389/fphar.2023.1343664

**Published:** 2023-12-18

**Authors:** Thet Thet Htar, Rakesh Naidu, Vuanghao Lim, Amit Kumar Pandey

**Affiliations:** ^1^ School of Pharmacy, Monash University Malaysia, Subang Jaya, Selangor, Malaysia; ^2^ Jeffrey Cheah School of Medicine and Health Sciences, Monash University Malaysia, Subang Jaya, Selangor, Malaysia; ^3^ Advanced Medical and Dental Institute, Universiti Sains Malaysia, Kepala Batas, Penang, Malaysia; ^4^ Department of Biotechnology, National Institute of Pharmaceutical Education and Research (NIPER-Ahmedabad), Gandhinagar, Gujarat, India

**Keywords:** breast cancer, small molecules, inhibitors, targeted therapy, drug design

Concern about the global burden of breast cancer is ever-growing with a projection of annual one million deaths by 2040. As breast cancer, is a highly heterogeneous type, the incidence and mortality rates are largely impacted by the type of treatment available for specific breast cancer subtypes. This eventually highlights the need for more personalized treatment and therapy. The landscape in breast cancer research has evolved over the past decades as we establish a growing understanding of cellular pathways and molecular targets. This has further accelerated the development of small molecule-based targeted therapy in oncology. Small molecule drug design enables tumor-cells specific death by binding to a target. In recent years there has been increasing interest in small molecule-based targeted drug development over macromolecular drug design, due to its advantages in pharmaceutical aspects. Majority of these small molecules targeted therapy in breast cancer are targeting signaling pathways (e.g., receptor tyrosine kinases), membrane receptors (e.g., EGFR inhibitors) and some regulatory proteins (e.g., PARP inhibitors). In view of recent developments, this Research Topic aims to spotlight the recent advances in small molecule-based targeted therapy for breast cancer. It comprises four literature reviews discussing new developments in targeted drug discovery and design in breast cancer.

Highlighting advancements, the review by Bansal et al. underscores the significance of tailored therapeutic approaches, including immunotherapy and targeted treatments, considering individual and tumor characteristics. Enzymes known as kinases, particularly their role in breast cancer initiation and progression, take center stage. Small-molecule kinase inhibitors, such as CDK4/6 and HER2 inhibitors, emerge as promising treatments, significantly improving overall survival in clinical trials. The review acknowledges challenges, including drug resistance and side effects, prompting ongoing research into innovative strategies like immunotherapies and precision medicine. Despite limitations, the review concludes by summarizing recent progress in small-molecule kinase inhibitors for breast cancer therapy, outlining current opportunities, challenges, and future research directions.

The review by Wu et al. highlights the promise of small-molecule tyrosine kinase inhibitors (TKIs), emphasizing their advantages in oral administration, multi-targeting, and low toxicity. Efforts to develop irreversible pan-HER TKIs, such as pyrotinib and neratinib, aim to overcome drug resistance. Clinical trials, including PHOEBE and NALA, demonstrate the efficacy of pyrotinib or neratinib combined with capecitabine in prolonging progression-free survival for HER2-positive metastatic breast cancer after multiple lines of anti-HER2 therapy failure. The review provides a comprehensive overview of the pathogenesis, standard treatments, mechanisms of irreversible TKIs, key trials, and the efficacy of irreversible TKIs in brain metastases, advocating for their use in combination with chemotherapy for HER2-positive metastatic breast cancer patients.

The systematic review by Chen et al. using a network meta-analysis made an interesting observation, demonstrating differences between different treatment regimens particularly the first-line treatments and third-generation EGFR TKIs for NSCLC patients with EGFR-sensitive and advanced EGFR-mutation in the Asian population. The authors’ findings revealed that furmonertinib (Fur), osimertinib (Osi) and aumolertinib (Aum) could be the most effective treatment options for Asian patients with locally advanced or metastatic NSCLC with EGFR-positive mutations with prolonged survival and reduced adverse side effects. The authors showed that the third-generation TKIs (Osi, Aum, and Fur) demonstrate significant benefits, improving progression-free survival (PFS) and overall survival (OS) in the first-line treatment compared with a first-generation (Gefitinib), and second-generation (Afatinib, Dacomitinib) TKIs. Combination of chemotherapy with TKIs also showed significant improvement of PFS and OS, specifically in combination with Gefitinib/Icotinib and Chemotherapy.

The authors, Zhu Z and Zhu Q, highlight that activation of several pathways could contribute to the resistance mechanism and differences between the cyclin-dependent kinase 4/6 inhibitors (CDK4/6i), Abemaciclib, Palbociclib, and Ribociclib. The differences in metabolic and transport mechanisms, and variations in chemical structures, preclinical pharmacology, and pharmacokinetics may affect the drug efficacy and safety. The findings of this review also suggest that genetic variations may influence the metabolism and response to CDK4/6 inhibitors, and personalized dosing strategies may be necessary to optimize treatment outcomes. The differences in their resistance mechanisms are one of the major concerns, and understanding these is important to improve patients’ outcomes and reduce serious side effects.

In summary, the rising impact of breast cancer worldwide calls for personalized treatments. The progress in small molecule-based therapies, especially kinase inhibitors, is encouraging for enhancing overall survival. Challenges like drug resistance and side effects persist, leading to ongoing exploration of innovative strategies. It highlights the need for tailored treatments and personalized dosing to achieve the best outcomes for patients.

